# mTOR Dysregulation, Insulin Resistance, and Hypertension

**DOI:** 10.3390/biomedicines12081802

**Published:** 2024-08-08

**Authors:** Silviu Marcel Stanciu, Mariana Jinga, Daniela Miricescu, Constantin Stefani, Remus Iulian Nica, Iulia-Ioana Stanescu-Spinu, Ileana Adela Vacaroiu, Maria Greabu, Silvia Nica

**Affiliations:** 1 Department of Internal Medicine and Gastroenterology, Carol Davila University of Medicine and Pharmacy, Central Military Emergency University Hospital, “Dr. Carol Davila”, 010825 Bucharest, Romania; silviu.stanciu@umfcd.ro (S.M.S.); mariana.jinga@umfcd.ro (M.J.); 2 Discipline of Biochemistry, Faculty of Dentistry, Carol Davila University of Medicine and Pharmacy, 8 Eroii Sanitari Blvd, 050474 Bucharest, Romania; maria.greabu@umfcd.ro; 3 Department of Family Medicine and Clinical Base, Central Military Emergency University Hospital, “Dr. Carol Davila”, 010825 Bucharest, Romania; constantin.stefani@umfcd.ro; 4 Surgery Department, Central Military Emergency University Hospital, “Dr. Carol Davila”, 010825 Bucharest, Romania; remus.nica@umfcd.ro; 5 Discipline of General Surgery, Carol Davila University of Medicine and Pharmacy, 8 Eroii Sanotari Blvd, 054474 Bucharest, Romania; 6 Discipline of Physiology, Faculty of Dentistry, Carol Davila University of Medicine and Pharmacy, 8 Eroii Sanitari Blvd, 050474 Bucharest, Romania; 7 Department of Nephrology, Faculty of Medicine, Carol Davila University of Medicine and Pharmacy, 020021 Bucharest, Romania; ileana.vacaroiu@umfcd.ro; 8 Emergency Discipline, University Hospital of Bucharest, 050098 Bucharest, Romania; silvia.nica@umfcd.ro; 9 Department of Emergency and First Aid, Carol Davila University of Medicine and Pharmacy, 8 Eroii Sanitari Blvd, 050474 Bucharest, Romania

**Keywords:** sedentarism, obesity, insulin resistance, protein kinases, mTOR dysregulation, metformin, inhibitors

## Abstract

Worldwide, diabetes mellitus (DM) and cardiovascular diseases (CVDs) represent serious health problems associated with unhealthy diet and sedentarism. Metabolic syndrome (MetS) is characterized by obesity, dyslipidemia, hyperglycemia, insulin resistance (IR) and hypertension. The mammalian target of rapamycin (mTOR) is a serine/threonine kinase with key roles in glucose and lipid metabolism, cell growth, survival and proliferation. mTOR hyperactivation disturbs glucose metabolism, leading to hyperglycemia and further to IR, with a higher incidence in the Western population. Metformin is one of the most used hypoglycemic drugs, with anti-inflammatory, antioxidant and antitumoral properties, having also the capacity to inhibit mTOR. mTOR inhibitors such as rapamycin and its analogs everolimus and temsirolimus block mTOR activity, decrease the levels of glucose and triglycerides, and reduce body weight. The link between mTOR dysregulation, IR, hypertension and mTOR inhibitors has not been fully described. Therefore, the main aim of this narrative review is to present the mechanism by which nutrients, proinflammatory cytokines, increased salt intake and renin–angiotensin–aldosterone system (RAAS) dysregulation induce mTOR overactivation, associated further with IR and hypertension development, and also mTOR inhibitors with higher potential to block the activity of this protein kinase.

## 1. Introduction

Sedentarism, obesity, smoking, older age and male sex are risk factors for diabetes mellitus (DM) and cancer [1]. Diets rich in refined carbohydrates, sugar and processed foods are correlated with an increased risk to develop metabolic syndrome (MetS), DM and cardiovascular diseases (CVDs) [2]. Globally, more than 425 million people have type 2 DM (T2D) [3], representing an alarming public health problem worldwide [4,5]. Globally, one of three deaths is attributed to CVD [6], and it is estimated that 1.56 billion people will develop hypertension by 2025 [7]. MetS also represents a serious health problem worldwide, being characterized by obesity, dyslipidemia, insulin resistance (IR), hyperglycemia and hypertension [8]. The prevalence of MetS in the adult population is between 15 and 25%, with an increasing number every year [9]. Moreover, visceral obesity together with a sedentary life and high-fat intake are important key factors for IR development [10]. Obesity and IR increase every year worldwide [11]. Obesity, especially visceral obesity, represents one of the major risk factors for human primary hypertension, accounting for 65% to 75% [12]. In addition, DM-obese patients with hypertension have a higher rate of developing chronic kidney disease (CKD) [13].

The mammalian target of rapamycin (mTOR) plays an essential role in regulating cell growth, proliferation, survival [14], protein synthesis [15], glucose and lipid metabolism [16]. Moreover, mTOR can regulate blood sugar levels [17]. mTOR dysregulation is correlated with various systemic pathologies including cancer, arthritis, osteoporosis and IR [18]. The main aim of this narrative review is to present the mechanism by which mTOR can induce the development of IR and, further, of hypertension.

## 2. mTOR Pathway Overview

mTOR is a serine/threonine protein kinase that belongs to the phosphoinositide 3-kinases (PI3K)-related protein kinase families, and is formed by two complexes known as mTOR complex 1 (mTORC1) and mTOR complex 2 (mTORC2) in mammalians [19]. Regarding the structure, both mTOR complexes have some similarities concerning the catalytic subunit, DEP domain-containing mTOR-interacting protein (DEPTOR), and the Tti1/Tti2. mTORC1 has six protein complexes, while mTORC2 has seven [20]. mTORC1 has three core components including mTOR, a regulatory protein associated with mTOR (Raptor), and mammalian lethal with Sec13protein 8, also known as GβL (mLST8) [21]. Moreover, mTORC2 core components include rapamycin-insensitive companion of mTOR (Rictor), stress-activated protein kinase-interacting protein 1 (MSIN1) and protein observed with Rictor 1 and 2 (PROTOR ½) [22]. mTORC1 core proteins include Raptor, mLST8, proline-rich protein kinase B (AKT) substrate (PRAS40) and DEPTOR [23]. Two small G proteins, Ras homolog enriched in the brain (Rheb) and Ras-related GTPases (Rags), are required for activation and localization of mTORC1. Moreover, Rags form two types of heterodimers, Rag-A or Rag-B with Rag-C and Rag-D [24]. 

Raptor and Rictor act as scaffolds for assembling the two mTOR complexes and as regulators for the binding of substrates [25]. mTORC1 activation occurs via nutrients and AKT, which phosphorylate the tuberous sclerosis complex 2 (TSC2) and inhibit it [26]. TSC refers to the altered gene of tuberous sclerosis complex, an autosomal dominant disease. TSC1 is an important component of the PI3K/AKT/mTOR pathway involved in cell growth, survival, proliferation and autophagy [27].

TSC1/2 regulate mTOR activity [28], and, therefore, their deletion reduces AKT activity [29], being negative regulators of the PI3K/AKT/mTOR signaling pathway together with phosphate and tensin homolog (PTEN) [30]. PTEN, TSC1 and TSC2 inhibit mTOR activity via PI3K/AKT inhibition [31]. Moreover, TSC mutations may affect glucose metabolism and insulin signaling, which can cause mTORC1 activation [32].

Growth factors, insulin, hypoxia, low ATP ratio, tumor necrosis factor alfa (TNF-α), Wnt, leucine, glutamate and arginine induce mTOR activation via different pathways [33], considered the central integrator of several signals [34] in both normal and pathological conditions [35].

**Figure 1:**
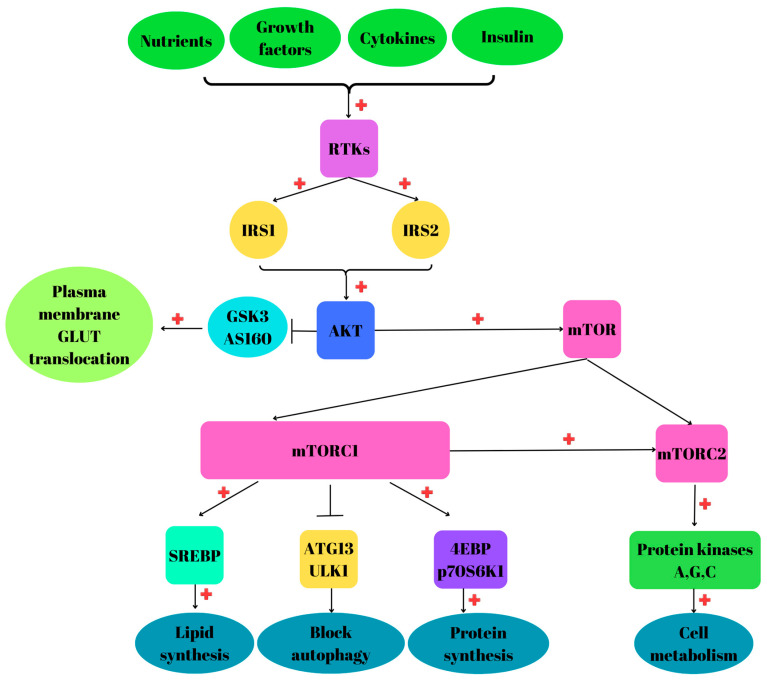
Phosphatidylinositol 3-kinase (PI3K) protein kinases B (AKT)/mammalian target of rapamycin (mTOR) pathway in healthy conditions: Nutrients, growth factors, cytokines and insulin bind to tyrosine kinases receptors (RTKs), leading to insulin receptors substrate 1 or 2 (IRS1/IRS2) activation and further AKT activation by phosphorylation. Once activated, AKT will phosphorylate other protein kinases such as mTOR, composed of the two complexes mTOR complex 1 (mTORC1) and mTOR complex 2 (mTORC2). mTORC1 activates sterol response element binding protein (SREBP) and eukaryotic translation initiator factor 4E binding protein (4EBP) and 70Ka ribosomal protein S56 kinase 1 (p70S6K1), leading to lipid and protein synthesis, respectively. mTORC1 inhibits the activity of unc-51-like kinase 1 (ULK1) and autophagy-related gene 13 (ATG13) blocking autophagy. Inactivation of AKT substrate 160 (AS160) and glycogen synthase 3 (GSK3) induces plasma membrane GLUT translocation. mTORC2 activates other protein kinases such as A, G and C, which positively regulate cellular metabolism. Activation of PI3K/AKT/mTOR will be correlated with cell growth, survival and proliferation. “+” activation.

Nutrients, energy, oxygen levels, stress and growth factors activate mTORC1 after their binding to tyrosine kinase receptors (RTKs) and further activation of PI3K and AKT, and inhibition of TSC1 and TSC2 [36]. Once activated by phosphorylation, AKT will exert a wide range of functions, such as cell growth, survival and metabolism [37]. AKT stimulates protein synthesis via mTOR activation, phosphorylates and inactivates AKT substrate 160 (AS160) and glycogen synthase kinase 3 (GSK3). Therefore, the inaction of AS160 and GSK3 will induce GLUT4 translocation into the plasma membrane, and glucose will enter the cells [38]. GSK3 is another important member of the PI3K/AKT/mTOR family involved in metabolism, proliferation, insulin signaling and apoptosis [39] (Figure 1).

Protein intake induces mTORC1 activation because amino acids stimulate the Rag GTPases to facilitate the binding to Raptor [40]. Insulin activates both mTORC1 and mTORC2 via PI3K [41]. Furthermore, mTORC1 activation can be performed by resistance exercises, leading to increased synthesis of muscle proteins [42]. Insulin-like growth factor (IGF-1R) and epidermal growth factor receptor (EGFR) regulate mTORC2 [43]. The presence of cytokines, nutrients, energy and hormones, in the cellular environment, induces the recruitment of mTORC1 from the cytoplasm to the lysosome via GTP-bound Rag-A or Rag-B and GTP-bound Rag-C or Rag-D [24].

mTORC1 directly phosphorylates 70 kDa ribosomal protein S6 kinase 1 (p70S6K1) and eukaryotic translation initiation factor 4E binding protein (4EBP) and induces protein synthesis. Moreover, mTORC1 stimulates lipid synthesis through the sterol response element binding protein (SREBP) transcription factor, which satisfies membrane formation during cell growth [44]. In addition, mTORC1 is located at the lysosomal surface, which controls protein synthesis and cellular growth. mTORC2 controls cell metabolism, survival and proliferation via phosphorylation of the protein kinases A, G, C and actin cellular cytoskeleton [45].

mTOR complex plays a key role in liver and adipose tissue lipogenesis via SREBP, considered the master transcriptional regulator of lipogenesis [46]. mTORC1 can phosphorylate various downstream effectors such as p70S6K and regulate mRNA translation [47] and cellular metabolic homeostasis [48]. mTORC1 inhibits autophagy by the phosphorylation of two kinases, unc-51-like kinase 1 (ULK1) and autophagy-related gene 13 (ATG13) [49].

The prolonged mTORC1 activation in the presence of a high-energy diet inhibits IRS via p70S6K blocking GLUTs inside the cells, increasing the blood glucose level, leading to T2D development [50]. In the case of DM patients, a significant downregulation of phosphorylated mTOR and p70S6K had been reported using peripheral blood mononuclear cells (PBMCs) [51].

## 3. Insulin Resistance Overview

In normal situations, after a meal, the increased plasmatic levels of glucose stimulate the secretion of insulin by pancreatic β cells, promoting carbohydrate uptake by adipose tissue, liver and skeletal muscles [52]. Therefore, in heathy conditions, insulin maintains the normal levels of blood glucose into the body organs by various mechanisms [53]. In the liver and skeletal muscle, insulin stimulates glycogen [54] and triglycerides synthesis [53]. Regarding the lipid metabolism in the adipose tissue, insulin stimulates fatty acids and triglycerides’ synthesis and inhibits lipolysis. In the liver and muscles, insulin decreases fatty acid beta-oxidation. The action of insulin in protein metabolism involves stimulation of protein synthesis in the adipose tissue, liver and muscle, by increasing the entry rate of amino acids into the tissue. In muscles, it reduces the degradation rate of proteins [55]. In the diabetic skeletal muscles, genes that encode carbohydrate, energy and amino acids pathways are decreased, including *IRS2*, *mTOR*, *SLC2A4* and *PPARA* [56].

In 1936, Himmsworth introduced the notion of IR, which divided diabetic patients into two categories: insulin sensitive and insulin-insensitive [57]. The incidence of IR in the Western population is around 25–35%, usually associated with obesity and obesity-related complications, being a risk factor for CVD, T2D, infertility, nonalcoholic fatty liver (NAFLD), certain types of cancer [58,59] and kidney dysfunction [60]. IR represents a metabolic disorder characterized by sensitivity to exogenous or endogenous insulin, leading to the body’s inability to capture glucose inside the cells [59]. Clinically, IR is also called IR syndrome or syndrome X [61], closely related to MetS [62], and is the first stage of T2D [63].

A very important aspect is that IR can be detected 10–20 years before the hyperglycemia’s clinical onset [64]. Moreover, IR can be predicted by several tests including blood glucose detection, serum insulin measurement, the glucose tolerance test and HOMA-R [65]. IR and hyperinsulinemia are characterized by higher levels of serum triglycerides and LDL, and decreased HDL [66]. Another important aspect is that elevated HbA1C between 7 and 10% is positively correlated with macro- and microvascular-T2D diseases [67]. IR is positively associated with visceral obesity, dyslipidemia, microalbuminemia and proinflammatory events [68]. In obese individuals, secretion of IL-1, TNF-α, leptin, adipsin, adiponectin and visfatin contribute to chronic inflammation development [69]. IR is also induced by the release of proinflammatory cytokines, such as TNF-α, with increased concentrations in chronic inflammation [70].

Systemic IR is characterized by ATP overproduction by several mechanisms including mitochondrial dysfunction, AMP-activated protein kinase (AMPK) inhibition, mTOR activation, hyperinsulinemia and hyperglucagonemia [71,72].

The liver, muscles and adipose tissue are affected by IR. Therefore, being insulin-dependent organs, glucose uptake is impaired. In addition, in the liver, insulin loses its capacity to inhibit glycogenolysis and gluconeogenesis [73]. IR is defined by the lack of receptors and GLUTs [74], and represents one of the main markers of T2D associated with lipid metabolism dysregulation. Because the skeletal muscle utilizes around 75–80% of systemic glucose, it presents a crucial role in IR [75]. Studies performed in humans and rodents reported that in skeletal muscles, IR decreases glucose uptake, inhibits glycogen synthesis, and induces hyperglycemia [76]. Additionally, IR and metabolic syndrome lead to muscle mass loss [77]. Thus, skeletal muscle-insulin resistance represents a major pathogenic factor for T2D or type 1 diabetes mellitus (T1D) [78].

In adipocytes, IR is characterized by decreased expression of GLUT4 and also dysregulation regarding secretion of leptin, TNF-α and adiponectin [79]. Adipose tissue-IR directly contributes to liver and muscle IR because higher levels of free fatty acids (FFAs) released would be delivered to these organs [80].

IR and IR syndrome may have several mechanisms as follows: a. genetic abnormalities regarding one or more proteins from the insulin cascade; b. fetal malnutrition; and c. visceral obesity [81]. Regarding T2D, several mechanisms have been postulated, including a lack of GLUTs, IRSs or glucose metabolism enzymes that did not interact with insulin receptors, leading to the inhibition of insulin transport and function [82].

T2D or IR lead to several complications, such as hypertension, atherosclerosis, liver disorders and various types of cancer [83].

## 4. mTOR Dysregulation and Insulin Resistance

Through its signaling cascade, insulin controls normal growth, metabolism and survival via the activation of PI3K, AKT, mTOR and Mitogen-Activated Protein Kinases (MAPKs). PI3K activation involves the activation of insulin receptor substrate 1 (IRS1), IRS2 and FoxO1 by phosphorylation, playing a key role in nutrient homeostasis and organ survival. IRS1 and IRS2 suppression by AKT inactivation leads to hyperinsulinemia and metabolic inflammation [84]. IRS1 is involved in glucose regulation, including GLUT4 translocation, while IR2 is implicated in adipocyte-fatty acid metabolism [85]. The insulin receptor is a tetramer protein that contains extracellular α subunits and transmembrane β subunits. Insulin or insulin-like growth factors (IGF-1s) bind to insulin receptor α subunits, leading to conformational changes of insulin receptors and dimerization of β subunits [86]. Therefore, IRS dysregulation is involved in metabolic disease initiation and progression [87].

Newgard and his research team reported that rats fed with a high-fat diet and a fat diet supplemented with branched-chain amino acids (BCAAs) develop IR. IR is correlated with chronic phosphorylation of mTOR, c-Jun N-terminal kinase (JNK) and IRS1 [88]. Additionally, BCAA overload leads to IR by mTOR activation [89].

Using primary cultures of rat cortical neurons, Pomytkin and his research team reported that elevated intracellular Ca^2+^ concentrations induced by glutamate and mitochondrial depolarization promote cytotoxicity on IRS, blocking them and further PI3K/AKT/mTOR, stimulating IR development [90].

**Figure 2:**
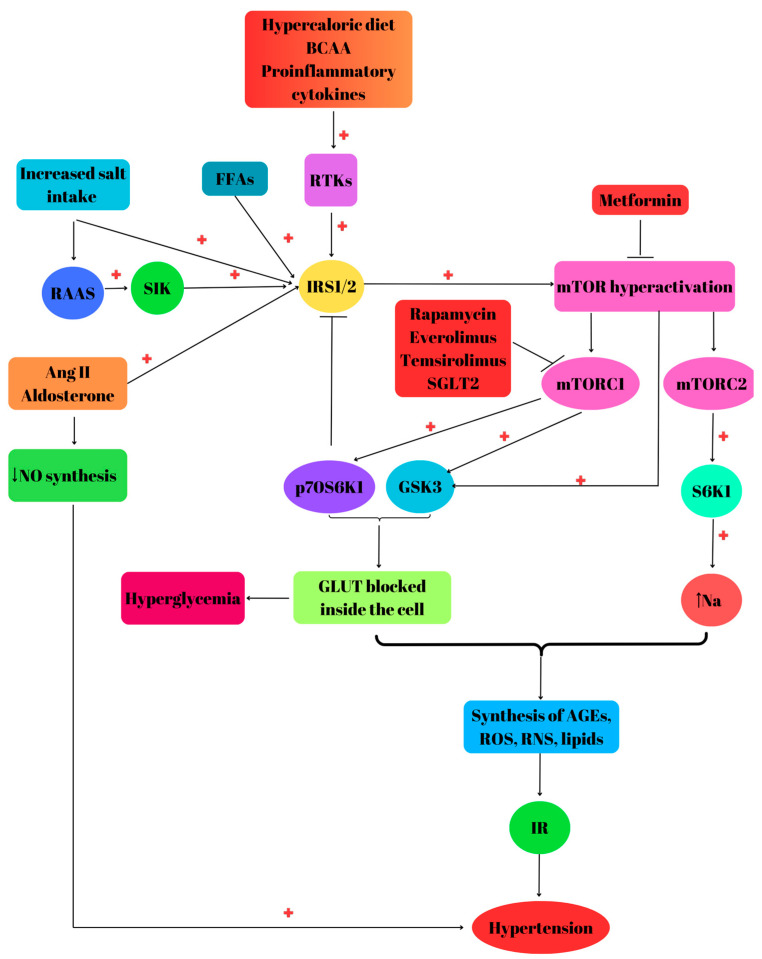
Phosphatidylinositol 3-kinase (PI3K) protein kinase B (AKT)/mammalian target of rapamycin (mTOR) pathway, insulin resistance and hypertension. Hypercaloric diet, branched-chain amino acids (BCAAs), proinflammatory cytokines, free fatty acids (FFAs), increased salt intake, the renin–angiotensin–aldosterone system (RAAS) and salt-inducible kinase (SIK) induce mTOR hyperactivation via RTKs or insulin receptor substrates (IRS1/2). IRS phosphorylation produced by angiotensin II (Ang II) and aldosterone decreases nitric oxide (NO) synthesis. Activation of 70Ka ribosomal protein S6 kinase 1 (p70S6K1) and glycogen synthase 3 (GSK3) inhibits IRS conducing to an increased blood glucose level because GLUT will be blocked inside the cell. mTOR complex 2 (mTORC2) activates serum/glucocorticoid-regulated kinase 1 (SGK1) stimulating Na transport. mTOR over-activation is associated with synthesis of advanced end products (AGEs), reactive oxygen species (ROS), reactive nitrogen species (RNS) and lipids. Metformin has the capacity to inhibit mTOR, while rapamycin, everolimus, temsirolimus and sodium-glucose transporter protein 2 (SGLT2) block mTORC1. All these events will lead to insulin resistance (IR) and further to hypertension. “+” activation; “↓” decrease; “↑” increase.

mTORC1 hyperactivation induces energy metabolism and translation dysregulation, leading to MetS [91]. Excessive consumption of glucose or BCAA chronically activates mTORC1, which further phosphorylates IRS1 at Ser 307 and activates S6K1. This aberrant activation decreases the activity and responsiveness of mTORC1 to insulin, leading to cell IR [92]. Dysregulation of the substrates for insulin receptors plays a key role in IR development, being controlled by more than 50 serine/threonine kinases, with positive or negative impact on insulin sensitivity [93]. In addition, overnutrition mediates obesity and triggers mTOR chronic hyperactivation in various tissues [94], promoting IR, hyperlipidemia, inflammation, stress and vasoconstriction [95]. Elevated FFAs contribute to IR by activation of IRS1 and further-downstream kinases such as mTOR, p70S6K1 and GSK3 [96]. Excessive alcohol consumption affects mTOR activity via dysregulation of protein synthesis and decreased activity of metabolic enzymes [97]. Long-term treatment with rapamycin disassembles mTORC2, leading to IR [98]. However, den Hartigh and his research team reported in a study conducted on obese male mice that a moderately low dose of rapamycin decreased weight gain and adiposity and improved the metabolic profile regarding triglycerides obesity [99]. Adipose tissue contributes to IR development, being an important source of proinflammatory molecule release such as TNF-α, leptin, IL-6, and anti-inflammatory compounds like adiponectin [100] (Figure 2).

## 5. Hyperglycemia, mTOR Dysregulation and Stress

At the beginning of the 20th century, the French chemist Louis Camille Maillard discovered the nonenzymatic and nonoxidative covalent attachment of glucose to proteins, lipids and nucleic acids. Glycoxidation refers to the radical reaction of free and protein-bound sugars. The Amadori rearrangement of the glycated proteins leads to AGEs generation [101]. AGEs can be found in T2D in elevated plasma levels and tend to accumulate in the tissue [89]. Hyperglycemia induces the activation of the polyol pathway, where the first and rate-limiting enzyme aldolase reductase (AR) reduces glucose to sorbitol, with NADPH as a donor of reduction equivalents. Sorbitol dehydrogenase oxidates sorbitol to fructose and NADH [102]. Further, fructose will be metabolized into ketone bodies, triose phosphate or will be transformed under the reaction catalyzed by fructose-3-phosphokinase in carbonyl compounds such as glyoxal, methylglyoxal and 3-deoxyglucose, with the formation of irreversible AGEs [103]. Methylglyoxal is one of the most potent glycating agents and leads to AGEs formation by endogenous, nonenzymatic glycoxidation of proteins, nucleic acids and lipids. Furthermore, methylglyoxal binds to arginine and lysine residues from proteins and also deoxyguanosine from DNA, resulting in the formation of AGEs and DNA adducts [104]. In vivo and in vitro studies revealed that methylglyoxal induces an inflammatory response, cytotoxicity and apoptosis [105]. In addition, the polyol pathway decreases the level of reduced glutathione (GSH) and other intracellular antioxidants because it uses NADPH and deprived glutathione reductase from it [106]. In chronic hyperglycemia, AGEs induce the development of macro- and microvascular complications in both T1D and T2D and enhance the expression of their cognate receptor, RAGE [107,108].

The hexosamine biosynthesis pathway (HBP) represents the link between glucose metabolism and lipid, nucleotide and amino acid metabolisms. Around 2–5% of glucose is converted via HBP to uridine diphosphate N-acetyl glucosamine (UDP-GlcNAc), used for glycolipids, glycoproteins and glycosaminoglycan synthesis, with the implication of fructose-6-phosphate aminotransferase as a rate-limiting enzyme [109]. Hyperglycemia activates the HBP pathway, which acts as an offshoot for glycolysis, leading to vascular damage. Moreover, in hyperglycemic conditions, the glycolysis intermediate fructose-6-phosphate is used to obtain UDP-GlcNAc, utilized as a substrate for acetylation of proteins at serine and threonine residues as O-linked N-acetylglucoseamines. This process of protein acylation is a reversible posttranslational modification that can alter the activity, interaction and function of targeted proteins [110]. Moreover, UDP-GlcNAc induces protein misfolding by interfering with *N*-linked glycosylation at the level of the endoplasmic reticulum (ER) membrane, leading to ER stress [111] and tumor progression [112].

**Figure 3:**
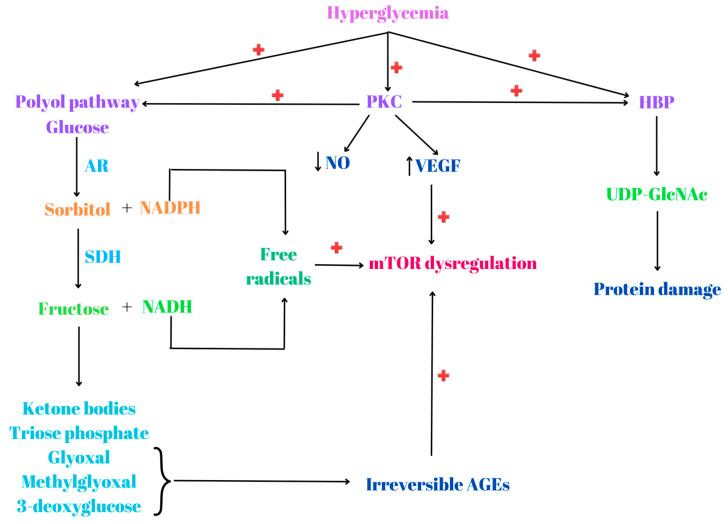
Hyperglycemia is correlated with the activation of the polyol pathway, where glucose is reduced to sorbitol by aldolase reductase (AR) and NADPH. Further, sorbitol will be oxidized to fructose by the enzyme sorbitol dehydrogenase (SDH), and NADH is generated. Fructose will be metabolized into ketone bodies, triose phosphate or carbonylic compounds such as glyoxal, methylglyoxal and 3-deoxyglucose. The last three compounds will contribute to irreversible advanced glycation end product (AGE) formation. NADPH and NADH represent sources for reactive species generation. In hyperglycemic conditions, the hexosamine biosynthesis pathway (HBP) is also activated, leading to the formation of uridine diphosphate N-acetylglucosamine (UDP-GlcNAc), which can induce protein damage. Protein kinase C (PKC) is activated by hyperglycemia, which has the capacity to activate polyol and HBP pathways. PKC activation is associated with decreased levels of nitric oxide (NO) biosynthesis and elevated levels of vascular endothelial growth factor (VEGF). All these molecular events will induce, in the end, mTOR dysregulation. “+” activation; “↑” increase; “↓” decrease.

Inflammation and oxidative stress (OS) are involved in diabetes pathogenesis, both in T1D and T2D, because free radicals damage pancreatic β cells. Sustained hyperglycemia leads to the formation of reactive oxygen species (ROS) and reactive nitrogen species (RNS) [113]. The mitochondrial electron transport chain, xanthine oxidase (XO), nicotinamide dinucleotide phosphate (NADPH) oxidase and uncoupled endothelial nitric oxide synthesis (eNOS) are the main ROS sources in diabetic blood vessels [114]. For example, the reaction of ROS with NO generates peroxynitrite (ONOO^−^), a powerful oxidant for DNA, lipids and cellular proteins. In this way, NO bioavailability is diminished, which causes endothelial dysfunction. In addition, in diabetes, activation of protein kinase C (PKC) via diacylglycerol (DAG), elevated flux of the HBP, the polyol pathway and increased levels of AGEs contribute to ROS production [115]. Higher levels of DAG activate the PKC pathway, inducing the formation of AGEs [116]. PKC activation is correlated with elevated endothelial inflammation, decreased levels of NO generation and increased expression of vascular endothelial growth factor (VEGF). Once activated, PKC activates MAPK and further PI3K/AKT/mTOR [117]. Taking into consideration all these molecular events, PI3K/AKT/mTOR dysregulation leads to IR, correlated with the synthesis of AGEs, ROS and RNS [88]. Moreover, AGEs, ROS and RNS formation induce stress, protein accumulation, Met S dysfunction and inflammation [90], inducing cell damage of proteins, lipids, sugars and nucleic acids [88] (Figure 3).

## 6. mTOR Dysregulation, Insulin Resistance and Hypertension

Normally, blood pressure represents the product of cardiac output and peripheral vascular resistance. When the systemic blood pressure decreases, kidney-juxtaglomerular cells produce renin, which activates liver angiotensin and produces angiotensin I (Ang I). Furthermore, Ang I will be converted into angiotensin II (Ang II) by angiotensin-converting enzyme (ACE) located in the lungs [118]. In addition, ACE can also be located in endothelial cells [119]. In insulin-target organs, it promotes nitric oxide (NO) and endothelin release, which produce vasodilation and vasoconstriction, respectively, improving glucose distribution [54]. In circulation, Ang II diffuses to the tissues and binds to its main receptors [119], stimulates blood vessel constriction and induces the release of aldosterone from the adrenal glands, leading to sodium reabsorption [118]. In aldosterone-sensitive distal nephrons, together with vasopressin, aldosterone will have synergic effects on the epithelial Na^+^ channels (ENaCs) to increase Na reabsorption [120].

Hypertension is often met in patients with hyperglycemia, IR and abdominal obesity. Therefore, diabetic patients will have a 2 to 4 times higher risk of developing CVD [121].

The renin–angiotensin–aldosterone system (RAAS) activation is the primary etiologic event in the development of hypertension in subjects with DM [122]. In humans, higher amounts of salt intake induce alteration of the RAAS system and further IR (Figure 2). Moreover, the release of Ang II from the salt-loading adipose tissue stimulates the synthesis of proinflammatory cytokines, lipogenesis and cardiac hypertrophy and reduces the synthesis of insulin. Studies performed on animals reported that a higher intake of salt leads to diabetes via RAAS activation and salt-inducible kinase (SIK). SIK is a serine/threonine protein kinase that may phosphorylate IRS1, leading to PI3K/AKT/mTOR activation [123].

RAAS leads to hypertension development because Ang II and aldosterone increase IRS phosphorylation at serine residue increasing the PI3K/AKT activity. This molecular event will be correlated with decreased activation of endothelial nitric oxide synthase, leading to reduced NO synthesis [124]. It is well known that insulin stimulates the production of NO from the vascular endothelium. IR induces a decreased production of NO, a key element for CVD development [125]. Endothelial tissue mediates vascular tone, cell growth and the interactions among thrombocytes, leukocytes and the vessel wall. Moreover, the endothelial tissue may synthesize growth factors and thrombo-regulatory molecules [126].

In the cardiovascular system and kidneys, a hypercaloric diet, together with aldosterone, and Ang II promote IR via mTOR-S6K1 activation [127]. IR increases the blood pressure by enhancing tissue Ang II and aldosterone activity and stimulates sympathetic nervous system activity and oxidative stress [126].

Aging is associated with structural and functional modifications of organs, which may lead to senescent endothelial cells, a risk factor for CVD, including hypertension and diabetes [128]. ENaCs play a crucial role in Na^+^ homeostasis; therefore, dysregulation leads to many forms of hypertension [129]. mTORC2 regulates serum/glucocorticoid-regulated kinase 1 (SGK1) activity, a key enzyme that stimulates Na^+^ transport via insulin and IGF-1 [130]. More than that, mTORC2 is implicated in the regulation of renal tubular Na^+^ and K^+^ transport [131]. When patients are subjected to pressure overload, the ventricular myocardium switches from fatty acid metabolism to glucose to provide energy. Moreover, subjects with contractile dysfunction of the heart are characterized by myocardial glucose metabolism perturbation leading to glucose-6-phosphate (G6P) accumulation. Further, G6P accumulation activates mTOR [132].

## 7. Metformin and mTOR Inhibitors

Antidiabetic medications have the property to reduce the oxidative stress induced by hyperglycemia [133]. Metformin is often used for the hypoglycemic effects in T2D. Besides its hypoglycemic property, metformin exerts anti-inflammatory, antioxidant, antitumoral [134,135,136,137,138], antiaging, hepatoprotective, cardioprotective and tissue regenerative effects [139]. Metformin is widely used in pre- and diabetic patients, and also as a PI3K inhibitor, because it has low toxicity [140].

Moreover, metformin inhibits hepatic gluconeogenesis and enhances the peripheral utilization of glucose. More than that, it is considered the first line of treatment against PI3K/AKT/mTOR inhibitors that induce hyperglycemia [141]. Metformin can inhibit respiratory mitochondrial complex I, called NADH: ubiquinone oxidoreductase. At the level of complex I, NADH is oxidized, after being formed in glycolysis, fatty acid β-oxidation and the Krebs cycle. In this way, metformin increases the accumulation of NADH leading to ROS formation, and decreases the synthesis of ATP [142].

Metformin also has the potential to inhibit the mTOR pathway by activating the AMPK, leading to tumor inhibition [143,144]. Moreover, in hepatocytes isolated from rats, at concentrations of 10 to 20 μM, metformin activates AMPK and decreases glycemia via inhibition of hepatic gluconeogenesis [139,145]. It was observed that the administration of metformin for a long term, such as 12 months, decreases the level of C-reactive protein (CRP) in both women and men with impaired glucose tolerance [146]. Furthermore, it increases the survival rate of diabetic patients with advanced pancreatic neuroendocrine tumors [147] and lymphoma [148].

Frequently, mTOR dysregulation is associated with the development of various types of cancer. Therefore, mTOR inhibitors have been tested in this aspect, representing powerful therapeutic agents [149]. In animal studies, Lamming et al. reported that rapamycin inhibits mTORC2, necessary for insulin to suppress hepatic gluconeogenesis [150,151]. Rapamycin analogs such as everolimus and temsirolimus can inhibit only mTORC1 [152]. It is well known that overactivation of mTOR is associated with IR, and caloric restriction and short-term treatment with rapamycin increase glucose uptake and insulin sensitivity [153].

Wang et al. investigated in vivo the effects of evodiamine on IR in obese animals. The study concluded that evodiamine could inhibit the phosphorylation of IRS1, leading to mTOR inhibition and improving glucose tolerance [154]. The results are contradictory because Pereira and his research team illustrated that the treatment of human adipocytes with rapamycin decreases glucose uptake, contributing to IR development [155].

Inhibition of p70S6K1 will improve the life of T2D patients, as it is a downstream effector of mTOR, which mediates glucose homeostasis, protein synthesis, RNA processing, cell growth and apoptosis [156]. Studies performed on knock-out model mice without S6K1 revealed that mTOR inhibition by rapamycin causes hypoinsulinemia and hypersensitivity to insulin [157].

Das and co-workers present new hopes regarding the effects of rapamycin in animal studies. The scientific group observed that treatment with an mTOR inhibitor decreases the level of glucose and triglycerides and reduces body weight. Moreover, the levels of lipid peroxidation were significantly reduced. Rapamycin can inhibit the phosphorylation of mTOR and S6K1 but not of AKT. Administration of rapamycin improves cardiac dysfunction in type 2 diabetic mice [158].

Another glucose-lowering agent, glucagon-like peptide-1 (GLP-1), exerts cardioprotective effects [159,160]. It has been observed that, in the proximal tubular kidney area, sodium-glucose transport protein 2 (SGLT2) inhibitors suppress the gene transcription for glycolysis, gluconeogenesis and the Krebs cycle, blocking the mTORC1 pathway [161].

It is already well known that rapamycin and everolimus inhibit mTORC1. Moreover, studies performed on animals revealed that rapamycin improves health. On the other hand, everolimus enhances immunity in elderly humans without causing side effects [162]. The study conducted on diabetic rats by Zhou et al. reported that rapamycin and metformin significantly ameliorated IR, decreased inflammation, blocked mTOR and induced autophagy [163]. Similar results were obtained by Reifsnyder et al. on type 2 diabetic mice regarding metformin and rapamycin [164], and also only with rapamycin [165].

Rapamycin reduces hyperglycemia, lipid peroxidation, endoplasmic reticulum (ER) stress and dyslipidemia and increases the antioxidant capacity in type 2 diabetic animal studies [166].

The treatment of patients with T2D with acute stroke with metformin also demonstrates its positive effects regarding the neurological function and oxidative stress-related markers [167].

The study performed by Temiz-Resitoglu using hypertensive male rats treated with rapamycin revealed that this mTOR inhibitor normalized the systolic blood pressure. In addition, the activity of ribosomal protein S6 was attenuated in the heart, aorta and kidney [168]. Wang and colleagues reported similar results regarding the effects of rapamycin on early cirrhotic portal hypertension rats [169]. Kumar et al. tested the inhibitory capacity of rapamycin on salt-induced hypertension and kidney injury using Dahl salt-sensitive rats and found that it improved the renal function but did not have good effects on blood pressure [170]. Besides animal studies, promising results come from in vitro studies, where extracts from some vegetables possess anti-obesity and anti-inflammatory properties, and also may block the activity of mTOR [171].

## 8. Conclusions

Today, diets rich in refined carbohydrates associated with sedentarism lead to obesity, dyslipidemia, IR and also hypertension. The PI3K/AKTmTOR signaling pathway exerts key roles in cell metabolism, controlling growth, proliferation and survival. Normally, mTOR activation can be performed by growth factors, insulin and cytokines that bind to RTKs, leading to AKT and mTOR activation. mTOR hyperactivation is induced by a hypercaloric diet, proinflammatory cytokines, BCAA and dysregulation associated with various systemic pathologies, including IR, cancer and CVD. mTORC1 activates p70S6K1, which will block the IRS conducting to hyperglycemia because GLUT will remain inside the cell. Increased salt intake activates the RAAS system and further PI3K/AKT/mTOR, leading to NO decreased production and hypertension development. In T2D patients, mTOR dysregulation will promote hyperlipidemia, inflammation and vasoconstriction. Metformin is an excellent drug with hypoglycemic, anti-inflammatory and antitumoral properties, also inhibiting mTOR. Studies performed on animals reported that metformin and rapamycin reduced inflammation, hyperlipidemia and hyperglycemia, and induced autophagy by blocking mTOR. Also, everolimus, temsirolimus and SGLT2 block mTORC1, with positive results regarding glucose sensitivity being reported in animal studies.

Taking into consideration all these aspects, mTOR overactivation plays a pivotal role in IR and hypertension pathology. Therefore, detection of mTOR together with other compounds from the PI3K/AKT signaling pathway is important for T2D and T1D patients. Administration of mTOR inhibitors to diabetic patients may decrease the complications of this pathology.
